# Rapid gamma oscillations in the inferior occipital gyrus in response to eyes

**DOI:** 10.1038/srep36321

**Published:** 2016-11-02

**Authors:** Wataru Sato, Takanori Kochiyama, Shota Uono, Kazumi Matsuda, Keiko Usui, Naotaka Usui, Yushi Inoue, Motomi Toichi

**Affiliations:** 1 Department of Neurodevelopmental Psychiatry, Habilitation and Rehabilitation, Graduate School of Medicine, Kyoto University, 53 Shogoin-Kawaharacho, Sakyo, Kyoto 606-8507, Japan; 2 Brain Activity Imaging Center, Advanced Telecommunications Research Institute International, 2-2-2 Hikaridai, Seika, Soraku, Kyoto 619-0288, Japan; 3 National Epilepsy Center, Shizuoka Institute of Epilepsy and Neurological Disorders, Urushiyama 886, Shizuoka 420-8688, Japan; 4 Faculty of Human Health Science, Graduate School of Medicine, Kyoto University, 53 Shogoin-Kawaharacho, Sakyo, Kyoto 606-8507, Japan; 5 The Organization for Promoting Developmental Disorder Research, 40 Shogoin-Sannocho, Sakyo, Kyoto 606-8392, Japan

## Abstract

Eyes are an indispensable communication medium for human social interactions. Although previous neuroscientific evidence suggests the activation of the inferior occipital gyrus (IOG) during eye processing, the temporal profile of this activation remains unclear. To investigate this issue, we analyzed intracranial electroencephalograms of the IOG during the presentation of eyes and mosaics, in either averted or straight directions. Time–frequency statistical parametric mapping analyses revealed greater gamma-band activation in the right IOG beginning at 114 ms in response to eyes relative to mosaics, irrespective of their averted or straight direction. These results suggest that gamma oscillations in the right IOG are involved in the early stages of eye processing, such as eye detection.

Eyes are the windows to the soul and thus are indispensable for human communication. When people detect the eyes of other individuals in the environment, they rapidly and automatically follow the attentional direction of averted eyes and feel/understand the internal states reflected in straight eyes. To explain such efficient processing of eyes, a previous psychological study theorized the existence of innate modules for the processing of eyes^1^. The initial process was proposed to be the detection of eyes in the environment, which subsequently activate other processes, such as recognition of eye direction. Consistent with this idea, behavioral studies have shown that even newborn infants can detect eyes and preferentially look at them compared with other stimuli^2^,^3^.

A number of previous neuroimaging studies have investigated the neural substrates of eye processing and reported that some brain regions, such as the superior temporal sulcus (STS), are more active in response to averted eyes than to straight eyes^4^,^5^,^6^,^7^,^8^,^9^,^10^,^11^,^12^. These studies focused on the processing of eye direction, which was assumed to be a secondary processing of eyes after the initial eye detection process. A few previous intracranial electroencephalogram (EEG) studies reported that the inferior occipital gyrus (IOG) was more active in response to eyes than to other types of stimuli such as flowers^13^,^14^. Because the IOG is the most posterior brain region that exhibits face-related activation, some researchers proposed that the IOG is involved in the initial stage of face processing, specifically, the processing of facial features^15^,^16^. Consistent with this idea, several neuroimaging^17^,^18^, intracranial EEG^19^, and stimulation^20^ studies confirmed that the IOG is involved in the processing of facial features. Because eyes are a facial feature, these data suggest that the IOG is involved in the initial stage of eye processing.

However, the temporal profile of eye processing in the IOG is not yet fully understood. Aforementioned intracranial EEG studies have conducted event-related potential (ERP) analyses and have reported that eyes versus other stimuli elicited higher amplitude in a negative deflection peaking at around 200 ms in the IOG^13^,^14^. Similarly, several scalp-recorded EEG studies reported that eyes, compared with other stimuli such as mosaics, elicited higher activity in a negative deflection peaking at about 170 ms at the posterior cortices^21^,^22^,^23^. However, a previous methodological study showed that ERP analysis primarily detects the low-frequency components of EEG data^24^. To fully elucidate high- and low-frequency neuronal activity at high temporal resolution, researchers must conduct time–frequency analyses^25^. A previous intracranial EEG study in which time–frequency analyses were conducted for IOG activity in response to faces found face-related gamma-band (higher than 30 Hz^26^) activity beginning at 110 ms^10^. Based on these data, we hypothesized that eye processing in the IOG may be initiated by gamma-band activity beginning at approximately 110 ms.

To test this hypothesis, we analyzed intracranial EEGs of the human IOG (Fig. 1) as the participants were presented with visual stimuli comprising only the eye region (Fig. 2). To investigate the effect of eyes, mosaic patterns were presented as control stimuli. To examine the effect of eye direction, averted and straight directions were prepared for both the eyes and mosaic stimuli. To further explore the effect of eye direction, a second stimulus in the opposite direction of the first (i.e., averted if the first gaze was straight or straight if the first gaze was averted) was presented 500 ms after the onset of the first stimulus. Previous behavioral studies have found that dynamic changes of eye direction are more ecologically valid and powerful than static direction^27^. Time–frequency statistical parametric mapping (SPM)^28^ and traditional ERP analyses were conducted. Because several lines of evidence revealed functional hemispheric differences during eye processing^29^,^30^, the IOG was analyzed in both hemispheres.

## Results

### Time–frequency SPM

The time–frequency maps of IOG activity for each hemisphere and each stimulus presentation were analyzed using the general linear model (GLM), including the effects of stimulus type and direction (Table 1).

Time–frequency clusters were considered to exhibit significant activation if they reached an extent (cluster size) threshold of *p *< 0.05 (family-wise error [FEW] corrected for multiple comparisons over the whole time–frequency space [0–500 ms and 4–300 Hz]), with a height threshold of *p* < 0.001 (uncorrected). Individual analyses with a height threshold of *p* < 0.05 (uncorrected) were also conducted and only the consistent effects among participants (≥80%) are reported.

First, to determine the effect of eyes, IOG activity was investigated during the first stimulus presentations (Fig. 3) using the contrast of eyes versus mosaics. In the right IOG, there was significant rapid and long-lasting gamma-band activity spanning 114–500 ms. Significant theta/alpha-band activity was detected from 0–289 ms; however, we do not discuss the temporal profile of this component further due to the time–frequency analysis limitation of time resolution for low frequencies. In the left IOG, the contrast revealed a small cluster of gamma-band activity at 225–233 ms.

Next, to determine the effect of eye direction, IOG activity was investigated during the first stimulus presentation using the contrasts of averted versus straight eyes and straight versus averted eyes. These contrasts did not detect any significant activation in either the right or left IOG. To investigate the effect of eye direction changes, IOG activity during the second stimulus presentations (Fig. 4) was tested using these contrasts. A small cluster of gamma-band activity was detected in the left IOG at 451–463 ms.

### ERP

ERP analyses were conducted on IOG activity to compare the current results with findings from previous studies^21^,^22^,^23^. The ERP for each stimulus presentation was analyzed using the GLM in the same manner as the above time–frequency SPM analysis (Table 2).

First, to investigate the effect of eyes, we examined the IOG activity in each hemisphere during the first stimulus presentation (Fig. 5) using the contrast of eyes versus mosaics. In the IOG of both hemispheres, the contrasts revealed significantly larger negative deflections peaking at about 170 ms, as well as larger deflections in later positive and negative components.

Next, to investigate the effect of eye direction, we examined IOG activity in each hemisphere during the first stimulus presentation using the contrasts of averted versus straight eyes and straight versus averted eyes. IOG activity during the second stimulus presentations was also examined using these contrasts to investigate the effect of eye direction changes. These contrasts did not reveal any significant clusters in either the right or the left IOG.

## Discussion

Our time–frequency analysis results demonstrated that the IOG displayed stronger gamma-band activation in response to eyes than to mosaics. Compared with the clear effect of eyes, an effect of eye direction was not evident in gamma-band activity in the IOG. The present findings are consistent with those of previous intracranial EEG studies reporting that the IOG was active in response to eyes, irrespective of gaze direction^14^. The present findings are also in line with those of previous neuroimaging^17^,^18^, intracranial EEG^19^, and stimulation^20^ studies indicating that the IOG is involved in the processing of facial features. The results also fit with a neuroscientific model suggesting that the IOG is involved in the initial stage of processing facial features and then forwards the information to other regions, such as the STS^15^.

More important, our results revealed that gamma-band activity in the right IOG in response to eyes versus mosaics occurred at 114 ms. This finding is inconsistent with those of an intracranial EEG study that conducted time–frequency analyses and reported null findings with regard to IOG gamma-band activity in response to eyes^13^. Methodological differences may account for the discrepancy in these results. For example, electrode placement was determined based solely on clinical criteria in both studies, and hence, may have covered functionally different regions of the IOG. The present finding is in line with that of an intracranial EEG study indicating that the IOG gamma-band activity at 110 ms is involved in the processing of facial features^19^; however, that study did not specifically investigate the processing of eyes. To our knowledge, this is the first study to suggest that eye processing is implemented as early as 114 ms by gamma-band activity in the IOG. These neural activation patterns may be interpreted using a psychological model^1^ such that the IOG activity reflects rapid detection processing for eyes, which then activates the processing of eye direction.

In the present study, rapid gamma-band activity related to the effect of eyes was evident only in the right IOG. This result is consistent with those of previous behavioral^29^,^30^ and neuroimaging^9^,^10^,^12^ studies showing right hemispheric dominance during the processing of eyes and extends them to the eye detection process. However, because the electrode placement in the present study was based on anatomical rather than functional information, further investigation is necessary to confirm whether there is hemispheric functional asymmetry regarding IOG activity related to eye detection.

Our results did not show any evident effect of eye direction and showed the effect of eye direction changes only during late stage (>400 ms) in IOG gamma-band activation. These results suggest that the IOG is not related to the rapid processing of eye direction. It is possible that the STS region regulates the rapid processing of eye direction and changes in eye direction, which has been suggested by several neuroimaging studies^31^, by processing eye information from the IOG in a feedforward manner and sending feedback information to the IOG.

Our results have several implications for the understanding of neural and psychological mechanisms involved in the processing of eyes. First, the results demonstrate that the IOG plays an important role in the processing of eyes. Although the IOG has been recognized as an important region in the face processing literature^15^, it has received relatively little attention in the eye processing literature^32^,^33^,^34^. Second, our results revealed that the neural activity related to eye detection occurred at about 110 ms, which refines the psychological model positing that the initial processing of eyes begins with eye detection^1^ based on highly specific temporal profiles. Finally, our results revealed that gamma-band oscillation was involved in eye detection, which adds to the emerging body of research indicating that gamma-band oscillations play important roles during cognitive processing^35^,^36^,^37^, including for social cognition^38^,^39^. In addition, because there is ample evidence suggesting that gamma-band oscillations are utilized during inter-regional communication^40^,^41^,^42^,^43^, the present results suggest that a series of eye-related processes may be implemented by the entrainment of gamma-band oscillations in the IOG and other brain regions. In summary, our results provide an important clue regarding the initial stage of psychological and neural processing of eyes with specific spatial, temporal, and frequency profiles.

Our ERP analysis results demonstrated that the bilateral IOG displayed larger negative deflections peaking at approximately 170 ms in response to eyes compared with mosaics. This result is consistent with those of previous intracranial^13^,^14^ and scalp^21^,^22^,^23^ EEG studies regarding the temporal profile, and suggests that one eye-related source of scalp-recorded ERP is located in the IOG. The temporal profiles of ERPs showing later activation than those for gamma-band activity are in agreement with those of previous scalp-recorded EEG studies, which demonstrated that ERPs at approximately 200 ms primarily reflected low- (e.g., theta) but not high- frequency (e.g., gamma) band oscillations^24^ and that transcranial magnetic stimulation in the occipital cortex first generated gamma-band activity and subsequently generated low-frequency band activity^44^. The different spatial profiles for the ERPs and gamma-band activity are also in line with findings from an intracranial EEG study showing that ERPs had broader activation relative to the localized gamma-band activation^45^. These differences in the temporal and spatial activation patterns across ERPs and gamma-band activity may be result of the participation of different neural populations at different frequencies^46^,^47^.

The present study has several limitations that should be acknowledged. First, because the electrode locations were chosen based solely on clinical criteria in this study, we did not record other brain regions related to eye processing, such as the STS and fusiform gyrus^12^. Therefore, the patterns of activation in these regions and their functional coupling with the IOG during the observation of eyes remain unknown. Future intracranial EEG studies evaluating these regions in clinical participants or reconstruction analysis of multiple sources from scalp-recorded data in normal participants^48^ would promote further understanding of the psychological and neural mechanisms of eye processing.

Second, mosaic images were used to control for low-level visual features (e.g., overall luminance) and directional information. Because the mosaics lacked multiple types of information that are typically found in eyes, the specific information that was related to the processing of eyes versus mosaics in the IOG remains unclear. Thus, the comparisons with different types of control stimuli, such as other facial features or non-face objects, will be an important consideration for future research.

Finally, it must be noted that the time–frequency analyses that involved wavelet decomposition in the present study are limited in terms of time resolution, even in the gamma-band. Wavelet decompositions smear the width of the peaks in original data, although the peaks can be estimated at the same latency^49^. Future studies that apply different analyses using techniques that have better time resolution than wavelet analyses, such as the Hilbert-Huang transformation^45^, may be able to identify more accurately the onset of gamma oscillations in the IOG that are related to eye information processing.

In summary, our intracranial EEG recording and its time–frequency analysis revealed greater gamma-band activation in the right IOG beginning at 114 ms in response to eyes relative to mosaics, irrespective of their averted and straight directions. These results suggest that eye detection is rapidly implemented by gamma oscillations in the right IOG.

## Methods

### Participants

Six patients (5 females and 1 male; mean ± *SD* age, 34.5 ± 7.9 years) participated in the present experiment. They were suffering from pharmacologically intractable focal epilepsy and were subjected to implantation of intracranial electrodes as part of their presurgical evaluations. The experiment was conducted 2.0–2.8 weeks after electrode implantation. Electrophysiological and surgical evaluations suggested that the main epileptic foci were in the hippocampus for five subjects and in the lateral temporal cortex for one subject.

Neuropsychological assessments confirmed that all participants’ language ability and everyday memory were intact. The intelligence quotient (IQ), measured by the revised Wechsler Adult Intelligence Scale, was in the normal range in five participants, and in the mildly mentally retarded range in one participant (mean ± *SD* full-scale IQ: 91.8 ± 19.2; mean ± *SD* verbal IQ: 86.7 ± 12.0; mean ± *SD* performance IQ: 100.7 ± 27.3). During the experiment, no seizure incidents were observed and all participants were mentally stable. All participants were right-handed, as assessed using the Edinburgh Handedness Inventory^50^. All had normal or corrected-to-normal visual acuity. All participants provided written informed consent after the procedure was explained fully. The study was approved by the ethics committee of Shizuoka Institute of Epilepsy and Neurological Disorders and was conducted in accord with the Declaration of Helsinki. The data from different electrodes were reported elsewhere^51^.

### Anatomical magnetic resonance imaging (MRI) assessment

Pre- and post-implantation anatomical assessments were conducted using structural MRI on a 1.5-T scanning system (Signa TwinSpeed, General Electric Yokokawa) using T-1 weighted images. Three-dimensional fast spoiled gradient-recalled acquisition was utilized with the following parameters: repetition time = 12 ms, echo time = 5 ms, flip angle = 20°, matrix size = 256 × 256, field of view = 22 × 22 cm, and 76 slices resulting in voxel dimensions of 0.8594 × 0.8594 × 2.0 mm thick. Pre-implantation MRI assessments and surgical evaluations did not reveal any structural abnormalities in the bilateral IOG of any participant.

Intracranial electrodes were implanted using the stereotactic method^52^, and the implantation sites were chosen based solely on clinical criteria. Subdural electrodes were implanted in the usual manner in both hemispheres for five participants and in the right hemisphere for one participant.

**Figure 1:**
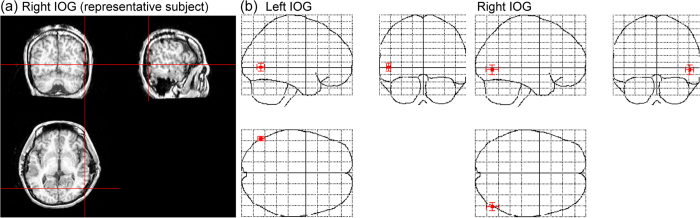
The location of electrodes in the inferior occipital gyrus (IOG). **(a**) Representative anatomical magnetic resonance images. The cross-hair indicates the location of electrodes in the IOG. (**b**) Mean (±*SD*) coordinates of the electrodes in the IOG in the Montreal Neurological Institute space.

The electrodes of interest in the IOG region were selected based on anatomical and functional criteria. First, post-implantation anatomical MRI assessments were conducted to confirm the positions of the electrodes. Individual MRI data were segmented into gray and white matter tissue to create a cortical surface rendering using the unified segmentation and normalization procedure^53^ in SPM8 (http://www.fil.ion.ucl.ac.uk/spm/) implemented in MATLAB 7.12 (MathWorks). The susceptibility artifact of each electrode was well visualized in the original MRI and in the surface-rendering image. One of the authors manually localized the electrodes in the IOG and measured the coordinates by viewing anatomical data using MRIcron software (http://www.mccauslandcenter.sc.edu/mricro/mricron/). This anatomical assessment showed that there were two candidate electrodes in each hemisphere in every patient. Then, we selected a single electrode from each hemisphere of each participant that showed clear face-related activity from 100–200 ms in an independent task^19^. To report the stereotactic coordinates of the electrode positions, the individual MRI was normalized to the standard stereotactic space defined by the Montreal Neurological Institute (MNI) using a unified segmentation and normalization procedure. The spatial transformation parameters from this normalization process were then applied to each of the electrode coordinates. The mean ± *SD* MNI coordinates of the electrode located in the IOG were as follows: left, *x* − 54.0 ± 3.6, *y* − 71.0 ± 5.2, *z* 1.3 ± 7.3; right, *x* 52.4 ± 6.4, *y* − 73.6 ± 9.1, *z* − 3.0 ± 8.8. Finally, the mean three-dimensional locations of the electrodes were projected on the MNI glass brain (SPM maximum intensity projection format; Fig. 1). The resulting six right and five left IOG electrodes were subjected to the following signal processing and statistical analyses.

### Stimuli

**Figure 2:**
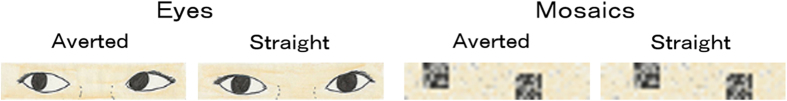
Schematic illustrations of stimuli. The actual stimuli were created using photographs of faces.

**Figure 3:**
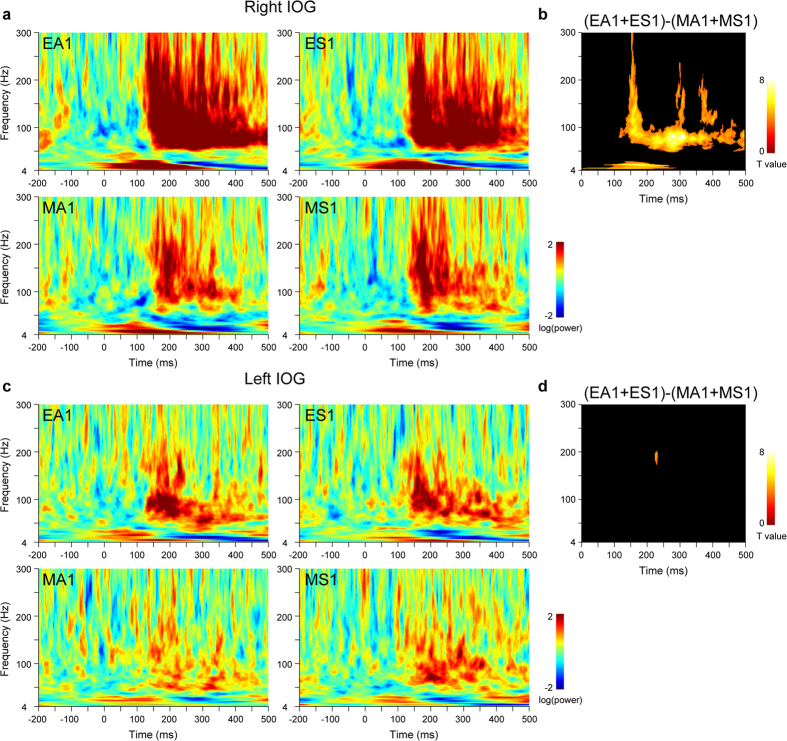
Time–frequency maps of activity in the inferior occipital gyrus (IOG) for the first stimulus presentations. (**a**) Time–frequency maps of right IOG activity. EA = averted eyes; ES = straight eyes; MA = averted mosaics; MS = straight mosaics; 1 = first stimulus presentation. (**b**) Statistical parametric maps for the contrast of eyes versus mosaics in right IOG activity. *p* < 0.05 cluster-level family-wise error (FWE)-corrected. (**c**) Time–frequency maps of left IOG activity. (**d**) Statistical parametric maps for the contrast of eyes versus mosaics in left IOG activity. *p* < 0.05 cluster-level FWE-corrected.

**Figure 4:**
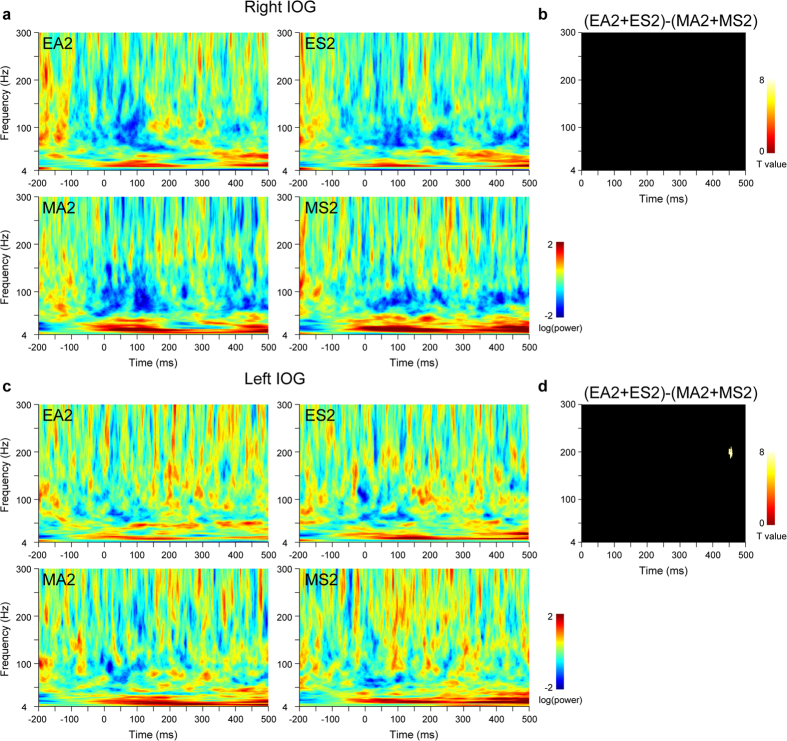
Time–frequency maps of activity in the inferior occipital gyrus (IOG) for the second stimulus presentations. (**a**) Time–frequency maps of right IOG activity. EA = averted eyes; ES = straight eyes; MA = averted mosaics; MS = straight mosaics; 2 = second stimulus presentation. (**b**) Statistical parametric maps for the contrast of eyes versus mosaics in right IOG activity. *p* < 0.05 cluster-level family-wise error (FWE)-corrected. (**c**) Time–frequency maps of left IOG activity. (**d**) Statistical parametric maps for the contrast of eyes versus mosaics in left IOG activity. *p* < 0.05 cluster-level FWE-corrected.

**Figure 5:**
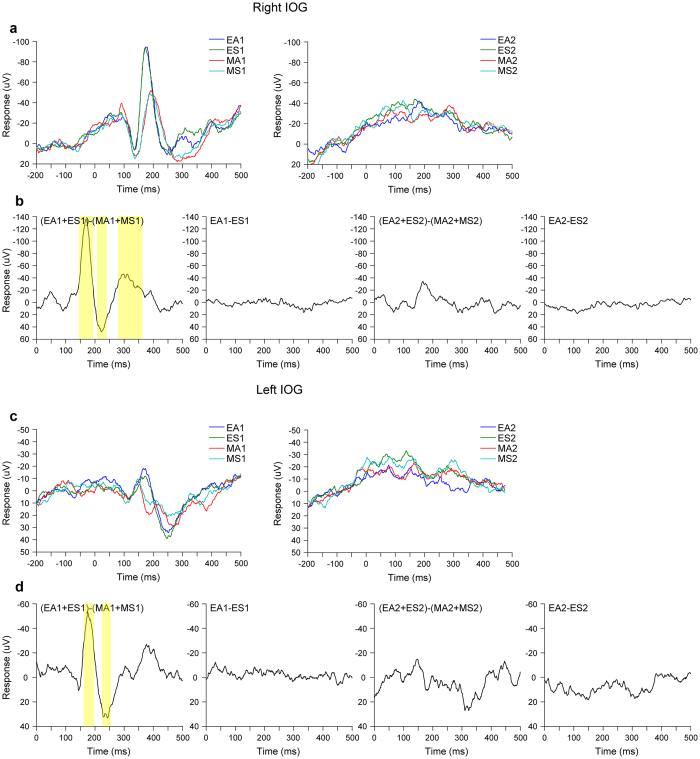
Event-related potential activity in the inferior occipital gyrus (IOG). (**a**) Grand-average ERP waveforms of right IOG activity. EA = averted eyes; ES = straight eyes; MA = averted mosaics; MS = straight mosaics; 1 = first stimulus presentation; 2 = second stimulus presentation. (**b**) Contrasted (differential) ERP waveforms of right IOG activity. Colored regions indicate significant differences. *p* < 0.05 cluster-level family-wise error (FWE)-corrected (two-sided evaluation). (**c**) Grand-average ERP waveforms of left IOG activity. (**d**) Contrasted (differential) ERP waveforms of left IOG activity. Colored regions indicate significant differences. *p* < 0.05 cluster-level FWE-corrected (two-sided evaluation).

Figure 2 illustrates the eye and mosaic stimuli, with the averted and straight directions indicated. Eye stimuli were prepared from color photographs of full-face neutral expressions of seven females and seven males, looking either to the left or straight ahead. Only the eyes were used from the photographs; no other facial features or eyebrows were visible in the stimuli. Mirror images of these stimuli were created using Photoshop 6.0 (Adobe). Eyes looking to the left or right were used for the averted-direction condition, and eyes looking straight ahead were used for the straight-direction condition. The mean luminance of the images was kept constant using MATLAB 6.5 (MathWorks).

The mosaic stimuli were constructed from the eye stimuli. First, all of the eye stimuli were divided into small squares (10 vertical × 50 horizontal), and all squares were set to the mean luminance of pixels in each square. To construct objects conveying directional information in a manner similar to the eye stimuli, two sets of 49 small squares with the highest luminance were selected and arranged randomly to construct two large diagonally aligned squares. The squares were aligned diagonally, because our preliminary experiment indicated that large squares arranged horizontally look like eyes. The horizontal center of these large squares was comparable to the pupil positions of the eye stimuli. Other small squares were then arranged randomly in other areas. These manipulations resulted in mosaic stimuli equivalent to the corresponding original eye stimuli in terms of overall luminance and directional information, without the incorporation of eye features.

Stimuli with different direction conditions were shown for the first and second stimulus presentations (i.e., averted after straight or straight after averted) to represent directional changes.

### Procedure

Stimuli presentation was controlled by SuperLab Pro 2.0 (Cedrus) and implemented on a Windows computer (FSA600, Teknos). Stimuli were presented on a 19-inch CRT monitor (GDM-F400, Sony) at a refresh rate of 100 Hz and a resolution of 1,024 × 768 pixels. Participants’ responses were recorded using a response box (RB-400, Cedrus).

Experiments were conducted individually in a quiet room. Participants were seated comfortably, with their heads supported by a chin-and-forehead rest positioned 0.57 m from the monitor. The resulting visual angle subtended by the stimulus was 1.5° vertically × 7.5° horizontally.

Each stimulus was presented three times. In addition, a red cross was presented as the target in 15 trials. Thus, each participant performed 183 trials: 42 trials each of averted eyes-straight eyes, straight eyes-averted eyes, averted mosaics-straight mosaics, and straight mosaics-averted mosaics, as well as 15 target trials. The stimuli were presented in a random order. In each trial, after the presentation of a cross-shaped fixation point for 500 ms, the first stimulus was presented for 500 ms in the center of the visual field. The second stimulus was then presented for 1,000 ms. In each target trial, instead of eyes or mosaic stimuli, the red cross was presented until a response was made. The participants were instructed to press a button using their right forefinger as quickly as possible after detecting the red cross. This task ensured that participants kept their attention on the stimuli, and it prevented the explicit processing of eye gaze. Performance on the target detection was perfect (correct identification rate = 100.0%), with no delay in reaction times (mean ± SD = 261.0 ± 15.6 ms). The post-hoc debriefing confirmed that the participants were not aware that the purpose of the experiment was to investigate gaze processing. The participants were instructed not to blink while the stimuli were being presented. Inter-trial intervals were varied randomly between 2,000 and 5,000 ms. To avoid habituation and drowsiness, participants were given short breaks every ~45 trials. Before data collection began, participants were familiarized with the procedure using a block of 10 training trials.

### Data recording

To examine cortical activity, intracranial EEGs were recorded using subdural platinum electrodes (2.3 mm diameter; Ad-tech). Depth platinum electrodes (0.8 mm diameter; Unique Medical) were also inserted to record subcortical activity (data not shown). Electrodes were referenced to electrodes (2.3 mm diameter; Ad-tech) that were embedded inside the scalp of the midline dorsal frontal region. Impedances were balanced and maintained below 5 kΩ. Data were amplified, filtered online (band pass: 0.5–300 Hz), and sampled at 1,000 Hz onto the hard disk drive of the EEG system (EEG-1100; Nihon Kohden). Online monitoring was conducted using a more restricted bandwidth of 0.5–120 Hz. Vertical and horizontal electrooculograms (EOGs) were simultaneously recorded using Ag/AgCl electrodes (Nihon Kohden). As in previous studies^54^, off-line visual inspection confirmed that the EOGs did not contaminate the intracranial EEGs. An unobtrusive video recording of events was made using the EEG system built-in video camera, and off-line analysis of the videos confirmed that all participants were fully engaged in the tasks.

### Data analysis: Preprocessing

Preprocessing, ERP analyses, and time–frequency SPM analyses were performed using SPM8 (http://www.fil.ion.ucl.ac.uk/spm) implemented in MATLAB R2012b (MathWorks).

Data obtained over 3,000 ms were sampled for each trial; pre-stimulus baseline data were collected for 1,000 ms, and experimental data were collected for 2,000 ms after stimulus onset at a sampling rate of 1,000 Hz. Epochs containing signals for which the amplitude was > ± 800 μV were excluded first, and then any epoch with an absolute signal amplitude value >5 SD from the mean or median signal amplitude for each electrode for each participant was rejected as an artifact. The frequencies of artifact-contaminated trials did not differ across conditions for either hemisphere (mean ± SD = 7.73 ± 2.91% and 8.27 ± 2.41% for the right and left IOG electrodes, respectively; p > 0.1, two-way repeated-measures analysis of variance).

### Data analysis: Time–frequency SPM analysis

Time–frequency SPM analyses^28^,^55^,^56^ were performed for IOG activity in each hemisphere. The time–frequency (power) maps were first calculated for each trial using continuous wavelet decomposition with 7-cycle Morlet wavelets during the whole epoch (−1,000–2,000 ms) and from 4 to 300 Hz, which covered theta (4–8 Hz), alpha (8–12 Hz), beta (12–30 Hz), traditional gamma (30–100 Hz), and high gamma (100–300 Hz) activities. The time–frequency maps were then cropped to −200–500 ms within the time period evaluated to prevent edge effects of the wavelet transformation. Finally, the time–frequency maps were log-transformed and baseline-corrected, with respect to mean power over the 200 ms pre-stimulus period, separately for each frequency. For analyses of the second presentation, the baseline included the period of the first stimulus presentations, as in previous studies that employed the consecutive presentations of eyes^57^,^58^,^59^ and faces^60^,^61^. This approach was used in the present study because the preliminary analyses for the second presentation using the baseline for the first stimulus revealed significant gamma-band activity for eyes versus mosaics immediately after stimulus onset (0 ms) and this activity was continuous with the activation of the first presentation.

The time–frequency maps were converted into two-dimensional images and then entered into the GLM based on a fixed-effects analysis of the pooled error from all trials of each hemisphere for all participants. Separate analyses were conducted for the first and second stimulus presentations. We set up a full factorial model including stimulus type (eyes or mosaics) and direction (averted, straight) as factors of interest. Corrections for non-sphericity (dependence and possible uneven variance between factor levels) were applied to ensure the assumption of an independent and identically distributed error for the GLM using the restricted maximum likelihood procedure^62^. The window of interest was restricted to whole frequency ranges (4–300 Hz) during the post-stimulus period (0–500 ms) using explicit masking. Finally, time–frequency SPM{*T*} data were calculated for each contrast.

Based on our interests, planned contrasts were performed for the comparison between eyes and mosaics [(averted eyes + straight eyes) - (averted mosaics + straight mosaics)], and the comparison between averted versus straight eyes (averted eyes - straight eyes; straight eyes - averted eyes) were tested for each hemisphere. Significantly activated time–frequency clusters were identified if they reached an extent threshold of *p* < 0.05 (FWE corrected for multiple comparisons over the whole time–frequency space [0–500 ms and 4–300 Hz] using random-field theory^63^,^64^) with a height threshold of *p* < 0.001 (uncorrected). Because the temporal resolution of low-frequency activity is generally poor in time–frequency analyses, the onset latencies are only discussed for high-frequency activity. To confirm the consistency of the effects among participants, additional individual analyses with a height threshold of *p* < 0.05 (uncorrected) were conducted; only robust (≥80%) effects in the individual-level analyses that reached significance in the above group-level factorial model analyses are reported.

Because there is debate regarding whether brain gamma-band activity can be contaminated by the electrical activities of ocular muscles^65^, preliminary time–frequency SPM analyses were conducted to assess the horizontal and vertical EOGs using the aforementioned fixed-effects analyses; no significant activity was observed for either the horizontal or the vertical EOGs.

### Data analysis: ERP

ERP analyses were conducted for IOG activity in each hemisphere. To analyze ERP, one-dimensional SPM analysis^66^,^67^, which is a variant of the three-dimensional sensor-space-time SPM approach^56^,^62^, was utilized. Because this study focused on a single electrode, the three-dimensional sensor-space-time SPM was reduced to the single-sensor-time SPM. Single trial responses from all trials for all participants were converted into one-dimensional line images after baseline correction for the −200–0 ms time period. The line images were then entered into the GLM in the same manner as in the above time–frequency SPM analysis. Planned contrasts and statistical inferences were also performed in the same manner as the above time–frequency SPM analysis.

## Additional Information

**How to cite this article**: Sato, W. *et al*. Rapid gamma oscillations in the inferior occipital gyrus in response to eyes. *Sci. Rep*. **6**, 36321; doi: 10.1038/srep36321 (2016).

**Publisher’s note:** Springer Nature remains neutral with regard to jurisdictional claims in published maps and institutional affiliations.

## Figures and Tables

**Table 1:** Time–frequency regions showing significant IOG activity.

Contrast	Hemisphere	Group-level^1^						Individual-level^2^
		Peak			Extent			
		Time (ms)	Frequency (Hz)	*T*-value	Time (ms)	Frequency (Hz)	Cluster size (ms × Hz)	No. of participants
Eyes - Mosaic (1st)	R	311	76	8.20	114–500	44–300	18548	6/6
		183	16	6.39	0–289	7–23	2438	6/6
		403	114	4.88	359–416	98–209	2309	6/6
		297	174	4.65	288–304	136–237	626	5/6
	L	228	189	4.16	225–233	171–201	209	5/5
Averted Eyes - Straight Eyes (1st)		none						
Straight Eyes - Averted Eyes (1st)		none						
Eyes - Mosaic (2nd)	L	460	199	3.91	451–463	184–210	186	5/5
Averted Eyes - Straight Eyes (2nd)		none						
Straight Eyes - Averted Eyes (2nd)		none						

R = right; L = left.

^1^*p* < 0.05, family-wise error-corrected cluster level.

^2^*p* < 0.05, uncorrected peak level.

**Table 2:** Event-related potential activity showing significant IOG activity.

Contrast	Hemisphere	Group-level^1^				Individual-level^2^
		Peak		Extent		
		Time (ms)	*T*-value	Time (ms)	Cluster size (ms× Hz)	No. of participants
Eyes - Mosaic (1st)	R	172	15.38	143–195	50	5/6
		313	6.16	280–365	86	6/6
		224	5.77	208–241	34	5/6
	L	177	6.69	163–198	36	4/5
		245	4.33	225–255	31	4/5
Averted Eyes - Straight Eyes (1st)		none				
Straight Eyes - Averted Eyes (1st)		none				
Eyes - Mosaic (2nd)		none				
Averted Eyes - Straight Eyes (2nd)		none				
Straight Eyes - Averted Eyes (2nd)		none				

R = right; L = left.

^1^*p* < 0.05, family-wise error-corrected cluster level.

^2^*p* < 0.05, uncorrected peak level.
